# Refractory pleural effusion in malignant hypertension leading to an unexpected diagnosis of tuberculosis: A case report

**DOI:** 10.3892/etm.2025.12978

**Published:** 2025-09-19

**Authors:** Hiroki Ito, Kentaro Yano, Yuya Suzuki, Yoshitaka Taniguchi, Fumiya Sato, Shigemitsu Sato, Takuo Hirose, Ikuko Oba-Yabana, Takefumi Mori

**Affiliations:** 1 Division of Nephrology and Hypertension, Faculty of Medicine, Tohoku Medical and Pharmaceutical University, Sendai, Miyagi 983-8536, Japan; 2 Division of Integrative Renal Replacement Therapy, Faculty of Medicine, Tohoku Medical and Pharmaceutical University, Sendai, Miyagi 983-8536, Japan

**Keywords:** malignant hypertension, tuberculosis, tuberculous pleuritis, acute kidney injury, pleural effusion

## Abstract

Accelerated malignant hypertension frequently manifests as multiple organ dysfunctions. However, persistent symptoms despite appropriate antihypertensive therapy warrant investigation of concurrent pathologies, particularly in patients with risk factors for opportunistic infections. A 57-year-old woman with untreated hypertension presented in August 2024, with markedly elevated blood pressure (208/122 mmHg), systolic dysfunction (ejection fraction, 42.5%) and acute kidney injury (creatinine 4.74 mg/dl). Accelerated malignant hypertension with multiple organ damage was diagnosed based on these findings. Despite optimal antihypertensive and diuretic therapy, pleural effusion and renal function progressively worsened. Thoracentesis revealed a lymphocyte-predominant exudative effusion with elevated adenosine deaminase levels. Subsequent investigations confirmed tuberculous pleuritis and peritonitis, ultimately diagnosed as miliary tuberculosis. Excessive diuretic therapy for presumed heart failure-related effusion exacerbates renal injury. Following initiation of antituberculous therapy, pleural effusion and renal function markedly improved. This case emphasizes the importance of reevaluating initial diagnoses when the clinical responses are suboptimal. In patients with multiple risk factors, particularly diabetes mellitus and kidney dysfunction, concurrent tuberculosis should be considered for treatment-refractory symptoms.

## Introduction

Accelerated malignant hypertension, a severe manifestation of hypertensive disease, is characterized by markedly elevated blood pressure (typically diastolic blood pressure >120-130 mmHg) and progressive target organ damage. This condition frequently leads to cardiac, renal and cerebral complications (1,2).

Tuberculosis remains a significant global health challenge. While Japan has seen a decline in incidence, the rate has plateaued in recent years, particularly among older adults, with >10,000 new cases still reported annually (3). Evidence increasingly suggests an association between tuberculosis infection and latent and cardiovascular risks, including hypertension (4-6). This association is particularly pronounced in patients with underlying conditions, such as diabetes mellitus and kidney dysfunction, which are known to impair immune function (7,8).

Tuberculous pleuritis, the most common form of extrapulmonary tuberculosis, represents a major cause of pleural effusion, accounting for ~5% of all tuberculosis cases globally (9). However, its diagnosis is often challenging when multiple pathologies coexist. Specifically, when organ dysfunction, for example heart or kidney failure, is attributed to primary conditions, such as accelerated malignant hypertension, a concurrent tuberculous infection can be easily overlooked (10).

The present report describes a case of refractory pleural effusion and acute kidney injury in a patient initially diagnosed with heart failure due to accelerated malignant hypertension. This case highlights the diagnostic challenge posed by coexisting pathologies and underscores the importance of considering tuberculosis in patients with treatment-refractory symptoms, with the aim of increasing clinical awareness for similar future cases.

## Case report

A 57-year-old woman presented to Tohoku Medical and Pharmaceutical University Hospital (Sendai, Japan) in August 2024, with progressive lower-extremity edema and uncontrolled hypertension. Despite awareness of hypertension since her 40s, the patient had received no treatment, with recent health screenings documenting systolic blood pressure consistently exceeding 200 mmHg. The patient's medical history was unremarkable, but the family history included hypertension in both parents, with a paternal history of type 2 diabetes mellitus and heart failure. The patient had a 37-pack-year smoking history.

**Figure 1:**
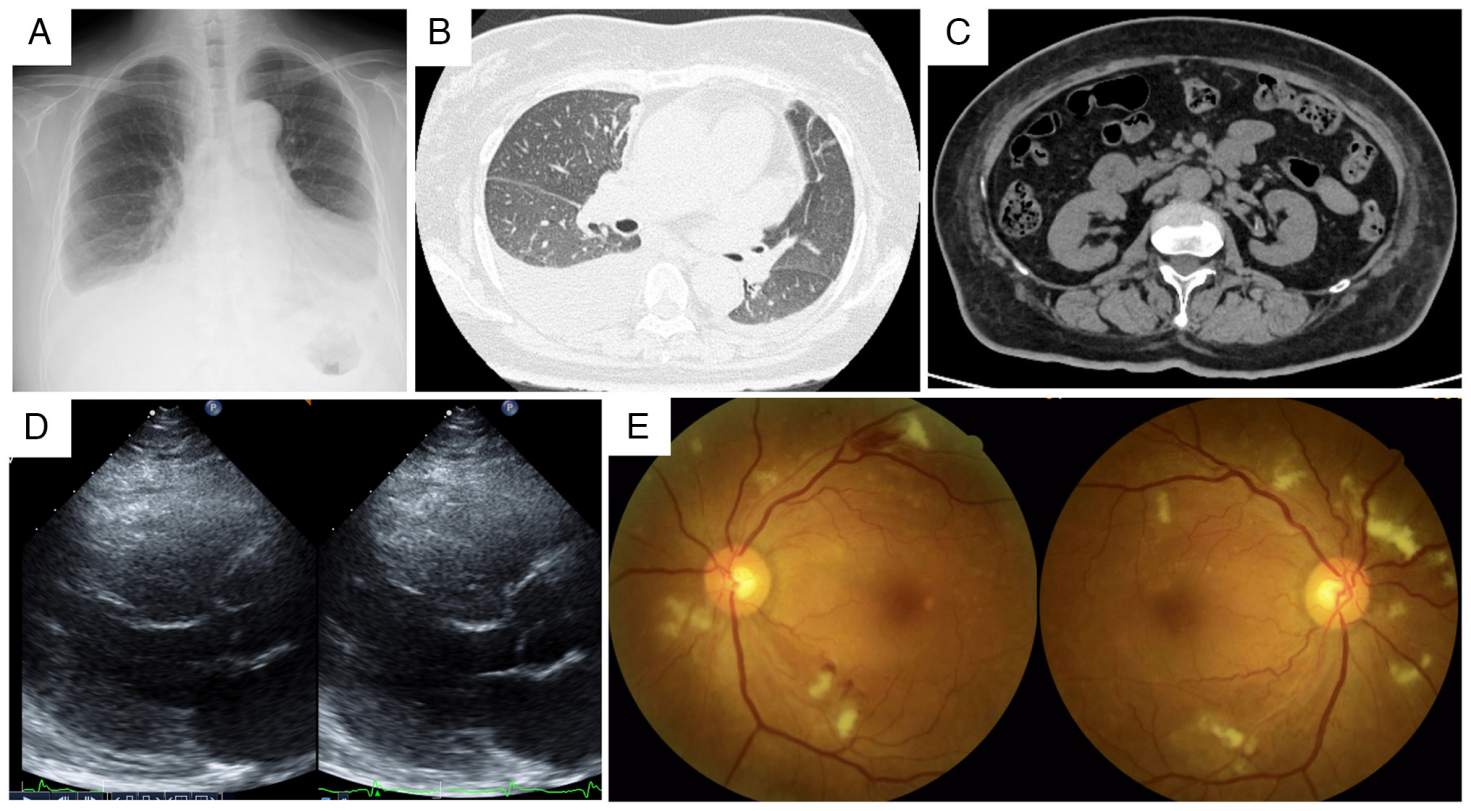
Radiological and imaging findings on admission. (A) Chest radiograph showing cardiomegaly with bilateral pleural effusion, predominantly on the right side. (B) Chest computed tomography image showing bilateral pleural effusion without significant parenchymal abnormalities. (C) Abdominal computed tomography showing subtle bilateral renal atrophic changes. (D) Transthoracic echocardiography revealing diffuse left ventricular hypokinesis with a reduced ejection fraction of 42.5%. (E) Fundoscopic examination confirming hypertensive retinopathy with hemorrhage and exudates.

Initial physical examination revealed a blood pressure of 208/122 mmHg (normal value, <120/80 mmHg), tachycardia (111 beats/min; normal range, 60-100 beats/min), a normal body temperature (36.5˚C; normal range, 36.0-37.0˚C) and mild hypoxemia (oxygen saturation, 93% on room air; normal value, ≥95%). Cardiovascular examination revealed a grade 3/6 systolic murmur at the second right sternal border, jugular venous distention and marked bilateral lower-extremity edema. Chest radiography and computed tomography revealed cardiomegaly, pulmonary congestion, bilateral pleural effusions with a right-sided predominance and bilateral renal atrophy (Fig. 1). Laboratory values demonstrated significant renal dysfunction (serum creatinine, 4.74 mg/dl; normal range, 0.6-1.0 mg/dl), moderate proteinuria (2.35 g/gCr; normal value, <0.15 g/gCr) and substantially elevated brain natriuretic peptide (3,252.6 pg/ml; normal value, <18.4 pg/ml). HIV test results were negative. C-reactive protein levels were minimally elevated at 0.87 mg/dl (normal value, <0.3 mg/dl) (Table I). Transthoracic echocardiography revealed diffuse left ventricular hypokinesis with reduced ejection fraction (42.5%; normal range, 55-70%) (Fig. 1D). Fundoscopic examination confirmed hypertensive retinopathy with hemorrhage and exudates (Fig. 1E).

**Figure 2:**
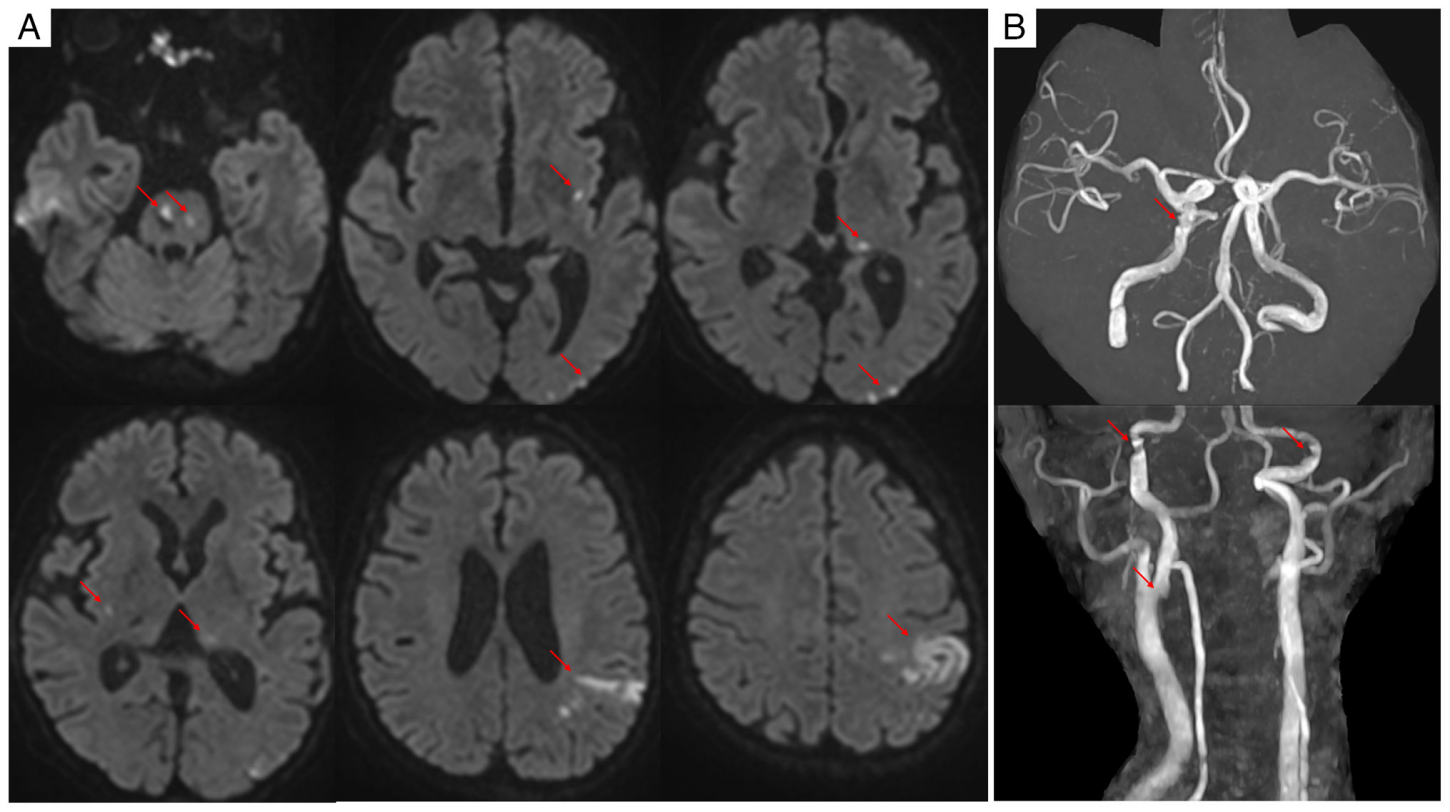
Neurological imaging findings. (A) Diffusion-weighted magnetic resonance imaging revealing multiple acute infarctions involving the pons, bilateral basal ganglia, left thalamus and left parieto-occipital region (arrow). (B) Magnetic resonance angiography showing vessel irregularities without obvious occlusion (arrow).

Heart and renal failure due to malignant hypertension were diagnosed based on these findings, and treatment with continuous nicardipine infusion (initiated at 2 µg/kg/min and titrated to maintain systolic blood pressure <160 mmHg) was initiated. Progressive dyspnea on minimal exertion and orthopnea prompted the administration of intravenous carperitide (0.025 µg/kg/min) and furosemide (80 mg/day). On hospital day 2, an altered mental status (Japan Coma Scale 3) (11) developed, characterized by word-finding difficulty, perseveration and a right-sided tactile extinction without apparent motor deficits (National Institutes of Health Stroke Scale 4) (12). Diffusion-weighted magnetic resonance imaging revealed multiple acute infarctions involving the pons, bilateral basal ganglia, left thalamus and left parieto-occipital region, with hemorrhagic transformation of the left parietal lesion (Fig. 2A). Magnetic resonance angiography showed vessel irregularities without obvious occlusion (Fig. 2B). Despite comprehensive evaluation, including carotid ultrasonography, Holter electrocardiography and malignancy screening, no embolic source was identified, leading to a diagnosis of embolic stroke of undetermined source (ESUS). Given concurrent renal dysfunction, therapeutic intervention was limited to aspirin (100 mg/day), with careful attention to blood pressure management.

**Figure 3:**
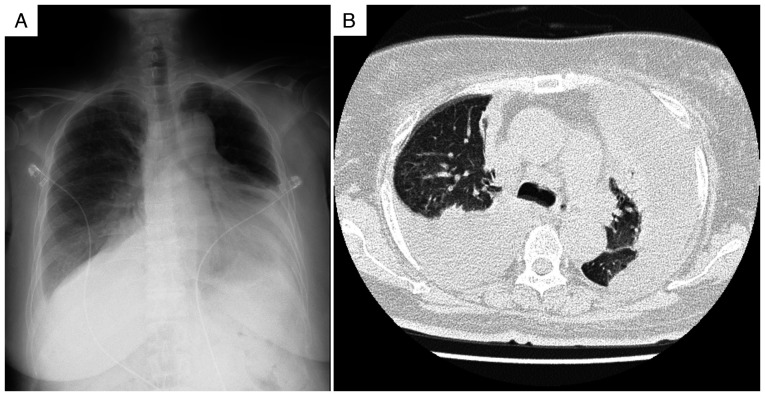
Radiological findings on hospital day 20, prior to the diagnosis of tuberculosis. (A) Chest radiograph showing persistent bilateral pleural effusions with notable progression of left-sided effusion. (B) Computed tomography revealing persistent bilateral pleural effusions despite optimal antihypertensive and diuretic therapy.

**Figure 4:**
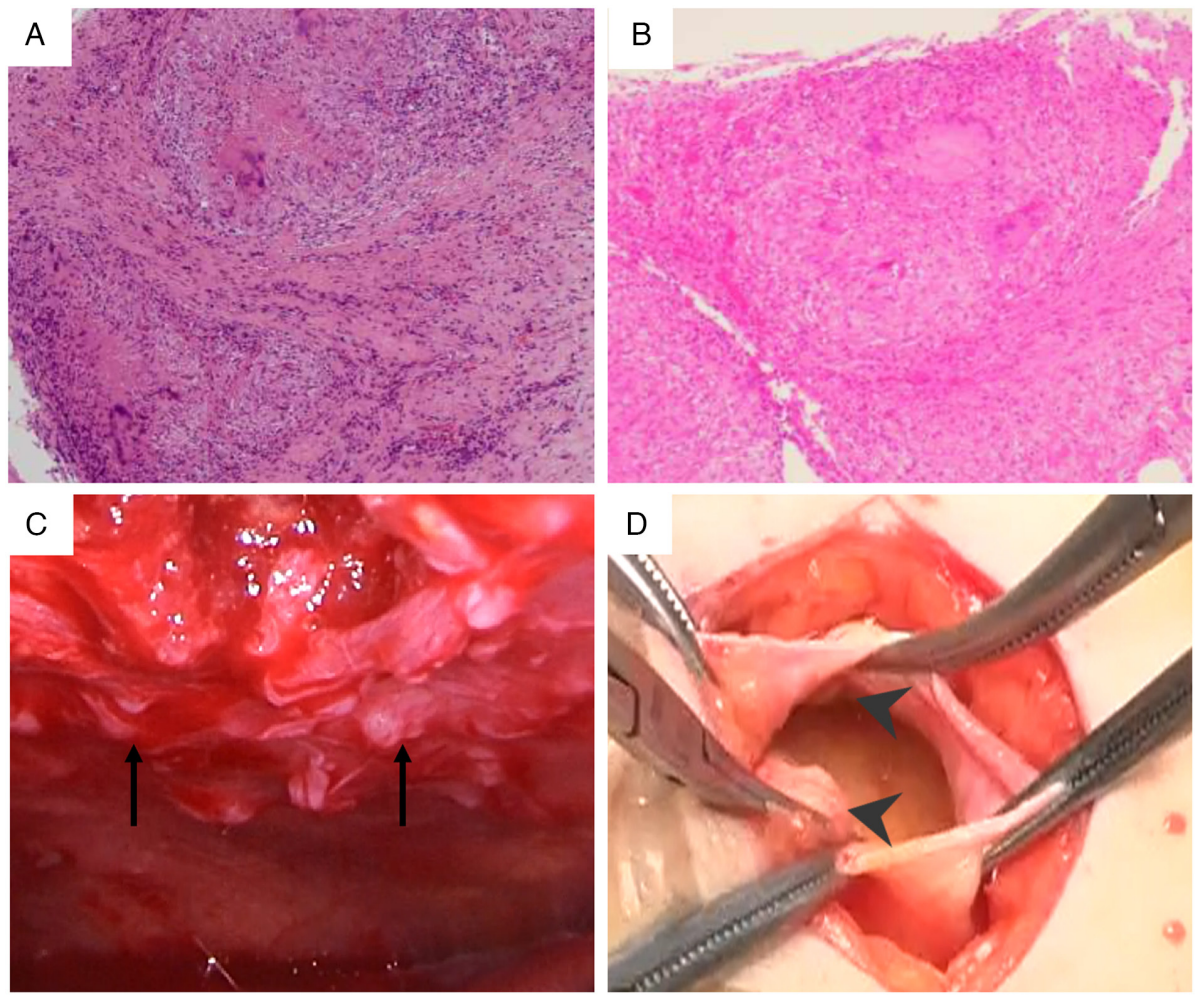
Thoracoscopic, intraoperative and histopathological findings confirming disseminated tuberculosis. (A) Photomicrograph of the pleural biopsy specimen showing a caseating granuloma surrounded by epithelioid cells and lymphocytes (H&E stain; magnification, x100). (B) Photomicrograph of a peritoneal nodule biopsy showing a caseating granuloma, confirming tuberculous peritonitis (H&E stain; magnification, x100). (C) Thoracoscopic view of the right pleural cavity, showing thickened pleura and multiple nodules (arrows indicate the nodules). (D) Intraoperative view during peritoneal dialysis catheter placement revealing multiple white nodules disseminated across the peritoneum (arrowheads indicate the nodules).

Persistent pyrexia and progressive elevation of inflammatory markers were observed. Despite adequate blood pressure control with multiple antihypertensive agents (including 80 mg/day nifedipine, 20 mg/day azilsartan and 10 mg/day carvedilol) and diuretics (15 mg/day tolvaptan, 40 mg/day furosemide and 25 mg/day spironolactone), pleural effusion and renal dysfunction persisted. As shown in Fig. 3, chest radiography revealed progression of the pleural effusions compared with that recorded at admission, and computed tomography confirmed large bilateral effusions with associated compressive atelectasis, indicating that the effusions were refractory to standard heart failure treatment. On hospital day 20, diagnostic thoracentesis was performed under ultrasound guidance. Approximately 800 ml of serous, straw-colored fluid was drained, and its analysis revealed lymphocyte-predominant (96%) exudative pleural fluid with elevated adenosine deaminase (ADA) levels (51.8 U/l) (Table II). A positive interferon-gamma release assay (IGRA; QuantiFERON-TB Gold Plus; Qiagen Inc.) result was noted, and although pleural biopsy showed no acid-fast bacilli on Ziehl-Neelsen staining, caseating granulomas confirmed tuberculous pleuritis. To establish a definitive diagnosis, a pleural biopsy was performed at 45 days of hospitalization. This biopsy confirmed tuberculous pleuritis by revealing caseating granulomas (Fig. 4A), although no acid-fast bacilli were seen. The procedure was guided by thoracoscopy, which had revealed multiple nodules on the pleura (Fig. 4C).

Peritoneal dialysis catheter placement was performed due to persistent elevation of serum creatinine (6-7 mg/dl). Although tuberculosis was suspected, a definitive diagnosis of tuberculous pleuritis remained pending, and multiple sputum cultures (three consecutive early morning samples) demonstrated no mycobacterial growth. Due to the progression of uremia requiring renal replacement therapy, a surgical procedure for peritoneal dialysis catheter placement was performed on hospital day 51. During the operation, visual inspection through the peritoneal incision revealed multiple disseminated peritoneal nodules. Subsequent histopathological examination confirmed caseating granulomas, establishing concurrent tuberculous peritonitis (Fig. 4B and D).

**Figure 5:**
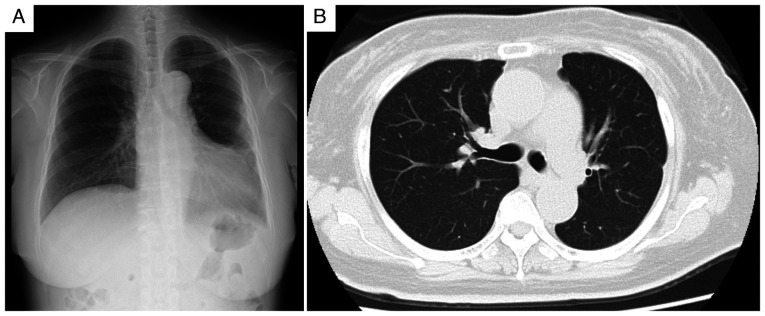
Follow-up radiological findings in May and June 2025, demonstrating sustained treatment response. (A) Chest radiograph showing sustained resolution of pleural effusion. (B) Chest computed tomography confirming the complete resolution of effusion and no new parenchymal abnormalities.

On hospital day 52, quadruple antituberculous therapy (300 mg/day isoniazid, 450 mg/day rifampicin and 1,200 mg pyrazinamide three times per week, and 750 mg ethambutol three times per week) was initiated and a marked improvement in pleural effusion was observed within 3 weeks of starting the therapy (Fig. 5A and B). Reduction of diuretic therapy, which involved discontinuing furosemide and reducing tolvaptan to 7.5 mg/day while spironolactone was continued at 25 mg/day, previously administered for presumed heart failure-related effusions, led to improved renal function, with serum creatinine decreasing to 2.61 mg/dl, and the peritoneal catheter was removed in October 2024. As of the last follow-up in June 2025, the patient's condition has remained stable with no recurrence of pleural effusion (Fig. 5A), with a serum creatinine level of 2.49 mg/dl. The patient continues to receive monthly follow-up care at the Outpatient Department, with ongoing management focused on controlling hypertension, managing chronic kidney disease and monitoring the patient for any recurrence of pleural effusion.

## Discussion

The present case illustrates a rare clinical scenario involving the coexistence of accelerated malignant hypertension and tuberculosis. The initial diagnosis of accelerated malignant hypertension with organ dysfunction was well-supported by the clinical presentation, including severe hypertension, cardiac dysfunction, acute kidney injury and hypertensive retinopathy (2,13). However, an inadequate response to standard therapeutic interventions provided crucial clinical indicators suggesting an additional underlying pathology.

Pleural effusion refractory to diuretic therapy warrants thorough investigation and illustrated a key diagnostic challenge in this case. Thoracentesis revealed lymphocyte-predominant (96%) exudative fluid with elevated ADA 51.8 U/l, findings strongly suggestive of tuberculous pleuritis (10,14). Although ADA levels >40 U/l are considered highly indicative (sensitivity, ~92%; specificity, ~90%), elevations can occur in other conditions and confirmation is often required (14,15). Acid-fast bacilli smear microscopy of pleural fluid demonstrates poor sensitivity (~5-10%) (9). Although the IGRA was positive, it cannot distinguish active disease from latent infection and has only moderate sensitivity (~77%) and specificity (~71%) for diagnosing tuberculous pleuritis, often necessitating additional testing (16). In the present case, a definitive diagnosis was established through pleural biopsy, which demonstrated caseating granulomas (9), highlighting the importance of pursuing invasive diagnostics when clinical suspicion remains high and noninvasive tests are inconclusive.

The systemic inflammatory response associated with tuberculosis likely contributed to endothelial dysfunction and vascular injury, thereby exacerbating the pathogenesis of malignant hypertension (4,17). Increasing evidence suggests a link between tuberculosis infection (even latent infection) and cardiovascular risks, such as hypertension (4-6). Tuberculosis infection activates monocytes and macrophages, enhancing the production of inflammatory cytokines (e.g., IL-6, TNF-α) that impair endothelial function by reducing nitric oxide availability and increasing vasoconstrictor production (6,18). Immunological changes, such as increased C-X3-C motif chemokine receptor 1 expression in activated monocytes, can promote endothelial adhesion and infiltration, thereby amplifying local inflammation (5). This persistent inflammatory milieu may have worsened vascular endothelial dysfunction and contributed to the severity of the hypertensive phenotype observed in the patient of the present study (5,19). Although the initial C-reactive protein level was only mildly elevated (0.87 mg/dl), it persistently remained >3 mg/dl prior to anti-tuberculous therapy, reflecting ongoing inflammation that likely contributed to the refractory nature of the patient's hypertension and organ damage. The interaction between tuberculosis and malignant hypertension appears to affect multiple organs, particularly the kidneys, potentially creating a vicious cycle (8).

The etiology of cerebral infarction in the patient of the present study was likely multifactorial. Accelerated malignant hypertension damages the cerebral vasculature through endothelial dysfunction and impaired autoregulation (2,13). However, the pattern of multiple acute infarcts across different vascular territories prompted the consideration of additional factors. Concurrent tuberculosis is increasingly being recognized as an independent risk factor for ischemic stroke, likely mediated by systemic inflammation, endothelial activation and potential prothrombotic effects (20-22). Although no embolic source was identified (ESUS classification), it was hypothesized that the synergy between severe hypertension-induced vascular injury and tuberculosis-mediated systemic inflammation significantly increases the propensity for these multiple cerebral events.

Progressive renal dysfunction provided additional diagnostic challenges. While hypertensive nephrosclerosis was initially considered, deterioration despite blood pressure control indicated the presence of other contributing factors. Aggressive diuretic therapy for presumed heart failure likely induced prerenal acute kidney injury (AKI) due to volume depletion. However, the marked improvement in renal function following anti-tuberculous therapy (serum creatinine falling from a peak of 7.11 to 2.61 mg/dl, obviating the need for renal replacement therapy) strongly suggested a direct contribution from the tuberculosis infection itself (23). Tuberculosis can impair renal function through several mechanisms, including direct bacillary invasion and granuloma formation within the kidney (renal tuberculosis), immune complex deposition leading to glomerulonephritis and systemic inflammation driven by cytokines affecting renal tissues (24,25). This case emphasizes the importance of considering tuberculosis in the differential diagnosis of AKI, particularly when other explanations are incomplete or the response to standard therapy is poor, as timely intervention can result in significant renal recovery (8,23). Patients with concurrent tuberculosis and severe renal impairment are at substantial risk, highlighting the necessity of accurate diagnosis and prompt management (8,23).

Multiorgan tuberculosis (pleuritis and peritonitis) was diagnosed incidentally during peritoneal dialysis catheter placement, highlighting the potential for disseminated disease, particularly in immunocompromised patients. Risk factors, notably uncontrolled diabetes mellitus (glycated hemoglobin A1c 7.4%) and kidney dysfunction, are well-established contributors to increased tuberculosis susceptibility (3,8). Diabetes mellitus is a major risk factor for active tuberculosis (7). These conditions likely facilitated the development and dissemination of tuberculosis in this patient.

In the present case, the coexistence of accelerated malignant hypertension and tuberculosis posed unique diagnostic and therapeutic challenges. Misattribution of pleural effusion to heart failure led to potentially deleterious therapeutic decisions, whereas the eventual diagnosis of tuberculosis necessitated a fundamental revision of the management strategy.

While this case report provides valuable clinical insights into a rare co-occurrence of diseases, it has several limitations. First, as a single case report, the generalizability of its findings is limited. The pathophysiological mechanisms observed (e.g., the association between systemic inflammation due to tuberculosis and the exacerbation of malignant hypertension) need to be validated in larger-scale studies. Second, although the individual patient's clinical course was meticulously examined, the patient's long history of untreated hypertension meant that the precise duration of hypertension and the specific details regarding treatment adherence during that period could not be fully ascertained in this case. Furthermore, definitively differentiating the causal relationship between tuberculosis and hypertension is difficult in a single case due to their complex interactions.

Future research should involve larger cohort studies to elucidate the pathophysiological mechanisms in the co-existence of tuberculosis infection and hypertensive diseases. In particular, detailed molecular-level investigations are warranted regarding the impact of chronic inflammation caused by tuberculosis on endothelial dysfunction and its role in the progression of multi-organ damage, including the kidneys. It is also crucial to consider when tuberculosis screening should be recommended for hypertensive patients, particularly those with risk factors such as diabetes mellitus and kidney dysfunction, including a cost-effectiveness analysis. As suggested by this case, establishing early diagnostic and interventional strategies for tuberculosis in patients with refractory pleural effusion and worsening renal function in the context of malignant hypertension could significantly contribute to improving outcomes in these complex cases.

In conclusion, this case highlights two crucial lessons: i) Treatment-refractory symptoms should prompt reevaluation of the initial diagnosis even when well-supported by clinical presentation; and ii) lymphocyte-predominant exudative effusions warrant investigation for tuberculous pleuritis, particularly in patients with risk factors such as diabetes mellitus and kidney dysfunction. Early recognition and appropriate intervention for concurrent tuberculosis can significantly improve outcomes in these complex cases.

## Figures and Tables

**Table I:** Laboratory data on admission.

Parameter	Result	Normal range
Urinalysis		
Protein	(3+)	Negative
Occult blood	(1+)	Negative
Red blood cells, /HPF	<1	<5
Protein content, g/gCr	2.35	<0.15
NAG, µg/l	10.7	<11.2
Blood cell counts		
White blood cells, /µl	9,000	3,300-8,600
Neutrophils, %	84.1	40.0-75.0
Hemoglobin, g/dl	11.9	13.0-16.0
Platelets, x10^4^/µl	33.4	15.0-35.0
Hematocrit, %	27.9	38.0-48.0
Blood chemistry		
Total protein, g/dl	6.9	6.7-8.3
Albumin, g/dl	3.4	4.0-5.0
Total bilirubin, mg/dl	0.85	0.3-1.2
AST, U/l	23	10-40
ALT, U/l	23	5-40
γ-GTP, U/l	59	10-70
HbA1c, %	7.4	4.6-6.0
Blood urea nitrogen, mg/dl	50	8-20
Creatinine, mg/dl	4.74	0.6-1.0
eGFR, ml/min/1.73 m^2^	8.2	>60
Glucose, mg/dl	132	70-109
Uric acid, mg/dl	12.4	3.5-7.0
TSH, µU/ml	2.9	0.5-5.0
Free T3, pg/ml	1.05	2.3-4.0
Free T4, ng/dl	1.24	0.9-1.7
Sodium, mEq/l	140	136-145
Potassium, mEq/l	4.2	3.5-5.0
Chloride, mEq/l	99	98-108
Calcium, mg/dl	8.9	8.5-10.2
Phosphorus, mg/dl	4.5	2.5-4.5
Iron, µg/dl	32	50-170
Ferritin, ng/dl	372	20-250
Total cholesterol, mg/dl	235	140-220
Triglycerides, mg/dl	150	30-150
LDL cholesterol, mg/dl	151	70-140
CRP, mg/dl	0.87	<0.3
IgG, mg/dl	1,533	870-1,700
IgM, mg/dl	44	35-220
IgA, mg/dl	492	110-410
C3, mg/dl	96	80-160
C4, mg/dl	33	15-45
CH50, U/ml	59.8	30-45
Anti-nuclear antibody (titer)	<1:40	<1:40
MPO-ANCA, U/ml	<1.0	<3.5
PR3-ANCA, U/ml	<1.0	<3.5
Anti-GBM antibody, U/ml	<2.0	<7.0
BNP, pg/ml	3,252.6	<18.4
Plasma renin activity, ng/ml	6.2	0.3-2.9
Aldosterone, pg/ml	71.5	40-310
ACTH, pg/ml	49.9	7.2-63.3
Cortisol, µg/dl	20.9	4.5-21.1
Metanephrine, mg/day	0.09	<0.5
Normetanephrine, mg/day	0.34	<0.9
Adrenaline pg/ml	38	<100
Noradrenaline, pg/ml	628	100-450
Dopamine, pg/ml	30	<30

HPF, high-power field; NAG, N-acetyl-beta-D-glucosaminidase; AST, aspartate aminotransferase; ALT, alanine aminotransferase; γ-GTP, γ glutamyl transpeptidase; HbA1c, glycated hemoglobin A1c; eGFR, estimated glomerular filtration rate; LDL, low density lipoprotein; CRP, C-reactive protein; IgG, immunoglobulin G; IgA, immunoglobulin A; C3, complement 3; CH50, complement hemolytic activity; MPO-ANCA, myeloperoxidase anti-neutrophil cytoplasmic antibody; PR3-ANCA, proteinase 3 anti-neutrophil cytoplasmic antibody; GBM, glomerular basement membrane; BNP, brain natriuretic peptide; TSH, thyroid-stimulating hormone; ACTH, adrenocorticotropic hormone.

**Table II:** Thoracentesis findings.

Parameter	Result	Normal range
Total protein, g/dl	4.8	<2.5
Albumin, g/dl	2.4	>1.2 (serum-pleural fluid albumin gradient)
LDH, U/l	204	<200
Cell count, /µl	2,936	<1,000
Neutrophils, %	2	<25
Eosinophils, %	0	<10
Basophils, %	0	<1
Lymphocytes, %	96	50-70
Monocytes, %	2	3-5
ADA, U/l	51.8	<40
CYFRA, ng/ml	14.6	<50
CEA, ng/ml	2.1	<5
ProGRP, pg/ml	168	<50
NSE, ng/ml	3.3	<15
pH	7.4	7.35-7.45
Glucose, mg/dl	131	70-109
Albumin, %	49.2	50-60
γ-globulin, %	18.5	11-22

LDH, lactate dehydrogenase; ADA, adenosine deaminase; CYFRA, cytokeratin fragment; CEA, carcinoembryonic antigen; ProGRP, pro-gastrin-releasing peptide; NSE, neuron-specific enolase.

## Data Availability

The data generated in the present study may be requested from the corresponding author.
